# New Insights into Tree Height Distribution Based on Mixed Effects Univariate Diffusion Processes

**DOI:** 10.1371/journal.pone.0168507

**Published:** 2016-12-21

**Authors:** Petras Rupšys

**Affiliations:** 1 Centre of Mathematics, Physics and Information Technologies, Aleksandras Stulginskis University, Kaunas, Lithuania; 2 Institute of Forest Management and Wood Sciences, Aleksandras Stulginskis University, Kaunas, Lithuania; Montana State University Bozeman, UNITED STATES

## Abstract

The aim of this paper is twofold: to introduce the mathematics of stochastic differential equations (SDEs) for forest dynamics modeling and to describe how such a model can be applied to aid our understanding of tree height distribution corresponding to a given diameter using the large dataset provided by the Lithuanian National Forest Inventory (LNFI). Tree height-diameter dynamics was examined with Ornstein-Uhlenbeck family mixed effects SDEs. Dynamics of a tree height, volume and their coefficients of variation, quantile regression curves of the tree height, and height-diameter ratio were demonstrated using newly developed tree height distributions for a given diameter. The parameters were estimated by considering a discrete sample of the diameter and height and by using an approximated maximum likelihood procedure. All models were evaluated using a validation dataset. The dataset provided by the LNFI (2006–2010) of Scots pine trees is used in this study to estimate parameters and validate our modeling technique. The verification indicated that the newly developed models are able to accurately capture the behavior of tree height distribution corresponding to a given diameter. All of the results were implemented in a MAPLE symbolic algebra system.

## Introduction

Understanding the key forces that shape tree heights distribution patterns and their dynamics through average breast height diameter within a forest stand (in the sequel–diameter) is a fundamental goal of forestry [1]. Stand volume, one of the most important variables in forest management, is heavily dependent on tree diameter and height distribution. The literature on forestry reports that tree height distribution varies across different stands and/or species. The tree height distribution is of prime importance from the point of view of the quality and quantity of a stand and its future growth. The importance of using the tree height rather than the tree diameter as a predictor of forest demographics arises from the former’s high potential for predicting the properties of forest productivity as pointed out by Kempes et al. [2]. Traditional methods quantify the tree size distribution in an even-age forest stand [3]. Unfortunately, height and diameter distributions cannot be combined if they are estimated independently using datasets from different stands. Much research has been conducted on the utilization of various theoretical functions for height and diameter distribution modeling techniques for improving stand volume prediction, such as Johnson’s [4–5], beta distribution [6], and power-normal [3]. Recently, there were also a few results published on the use of the copula approach for the modeling of tree height and diameter distribution in stands [7], [8].

The relationship between height and diameter varies for the same tree in different forest stands, such that there is a distribution of tree heights for any given tree diameter based on environmental conditions, or a random site effect. The different height-diameter relationships affect growth predictions and stand trajectories. The new developed stochastic differential equation (SDEs) based modeling approach for complex stands uses stochastic height-diameter relationships at the individual-tree level representing tree growth and neighborhood interactions that are then aggregated to predict the stand height structure. In this study, to project height distribution for a given diameter, a one-dimensional SDE with mixed effects was employed. The main feature of mixed effects models is that they allow parameter vectors to vary from plot to plot by splitting regression coefficients into a fixed part, common to the population, and random components, specific to each plot [9]. Mixed effects models allow fixed and random parameters to be estimated simultaneously and evaluate the value of the random parameters for a location not present in the original estimation dataset. This approach is known as calibration and can be applied if a sub-sample of trees measured for the total height and breast height diameter are available [10]. Fixed effects parameter SDEs are used in a wide range of applications in environmental, engineering, and biological modeling [11–14]. Discrete stationary stochastic models defined by Markov chains have been used to describe size-structure predictions [15].

The essential features of developed height distributions for a given diameter may be explained as follows. Heights are measured at different diameters in a number of sample plots. The diameters and number of measurements differ among plots and the measurements of the diameters are not evenly spaced. The diameter based dependent tree height distribution models are assumed to have some fixed effects parameters that are common to all plots and random effects that are specific to each plot. Two sources of variation were simultaneously included for modeling tree height distribution: variability between plots using a random effects approach and variability in the individual tree height using system noise, which reflects the random fluctuations around the corresponding theoretical height-diameter model. New developed conditional probability density functions of a tree height at a given diameter based on diffusion processes can be used for calculating the mean value of growth and yield attributes and its coefficient of variation as a function of tree height at any specified diameter. The random effects SDEs height-diameter relationships allow taking into account the effect of multiple causal relations in the model, the influence of unknown covariates affecting the height growth and they allow for developing height distribution accounting for spatial variability in large-scale modeling.

In this study, the evolution of a random variable (height), H(d), for a given diameter, d, is modeled using mixed effects SDEs from the Ornstein—Uhlenbeck family [16], for example, the Vasicek, the Gomperz (3-parameters and 4-parameters), the Bertalanffy, and the Gamma. We focused on mixed effects SDEs with a deterministic term depending on random effects and a stochastic term without random effects.

The aim of this study is to present the advantages of SDEs with mixed effects in analyzing tree height distributions for a given diameter and their application to describe the evolution of the height-diameter ratio, quantile curves, mean tree height, mean stem volume and the coefficients of variation for the mean tree height and stem volume. We also discuss how a conditional height’s probability density function for a given diameter can be used to construct maximum likelihood estimators using large collections of datasets provided by the Lithuanian National Forest Inventory (LNFI). A MAPLE program was used to carry out the calculations.

## Materials and Methods

A typical diffusion process is modeled as a differential equation involving a deterministic (drift) term and a stochastic (diffusion) term, the latter represented by Brownian motion [14]. Traditionally used ordinary differential equation models are the Malthus, Mitscherlich, Gompertz, and Bertalanffy types [11–13] (see Appendix A).

There are alternative ways of introducing stochasticity in an ordinary differential equation. In this work, the tree height randomness was approximated as a standard Brownian motion [11–14]. Therefore, the complete deterministic models defined by Eqs A.1, A.3, A.4, A.7 and A.9 were converted, into stochastic models assuming that the deterministic parameter, *α*, varies randomly around the mean:

$$\alpha (d)=\left\{ \begin{array}{l} \alpha +\sigma \varepsilon (d),\;\text{for Eqs}.\;\text{C}.1,\;\text{C}.3,\;\text{C}.4,\\ \frac{\alpha \beta \gamma }{e^{\beta d}-\gamma }+\sigma \varepsilon (d),\;\text{for Eq}.\;\text{C}.7,\\ \frac{\alpha }{d}-\beta +\sigma \varepsilon (d),\;\text{for Eq}.\;\text{C}.9. \end{array}\right.$$ (1)

where *σ* (*σ>0*) is the diffusion coefficient, which reflects random fluctuations around the corresponding theoretical height-diameter curve, and *ε*(*d*) is a Gaussian white noise process. If the magnitude of the parameter capturing system noise, σ, is zero, the entire system noise term will vanish, and the remaining part of the SDEs will simply be differential forms, the solutions to which are Eqs A.2, A.5, A.6, A.8 and A.10, respectively.

The relationships between total tree height and diameter are altered by environmental conditions. Among other plot-specific characteristics such as soil type, nutrient status, resistance of trees to windthrow, competition for light, and elevation cause the parameters to differ across plots. In the case of between-plot variations, the fixed effects parameters *α*, *β*, and *σ* vary from plot to plot and, hence, account for these variations. For the construction of the mixed effects parameters models, the first step is to determine which parameters should be considered mixed effects and which should be considered purely fixed effects. The parameters with high variability could be considered mixed effects. The parameter *α* has high variation between plots for all used SDEs models, so it can be altered by adding plot-specific random effects to the fixed effects parameter to produce a plot-specific parameter in the following form:

$$\alpha +\phi_{i},$$ (2)

where *ϕ*_*i*_ (*i = 1*, *2*, …, *M*)—plot-specific random effects, *M* is the number of plots. It is assumed that the random effects, *ϕ*_*i*_, *i = 1*, *2*, …, *M*, are independent and normally distributed with *0* mean and constant variance $\sigma_{\phi }^{2}$ ($\phi_{i}∼N\left(0;\sigma_{\phi }^{2}\right)$).

In order to derive mixed effects SDEs height-diameter models, it is sufficient to substitute Eqs 1 and 2 into Eqs A.1, A.3, A.4, A.7 and A.9. In this study, the tree height, H^i^(d), *i* = 1,2,…,*M*, evolving in M different experimental plots randomly chosen from a theoretical population was described by the Itô [17] sense SDE of the Vasicek type:

$$dH^{i}\left(d\right)=\beta \left(\left(\alpha +\phi_{i})-H^{i}(d\right)\right)\cdot dd+\sigma \cdot dW^{i}\left(d\right),\;P\left(H^{i}\left(0\right)=1.3\right)=1,\;d\in \left[0;D_{0}\right],$$ (3)

the 3-parameters Gompertz type:

$$dH^{i}\left(d\right)=\left[\left(\alpha +\phi_{i}\right)H^{i}(d)-\beta H^{i}(d)\ln(H^{i}(d))\right]\cdot dd+\sigma H^{i}(d)\cdot dW^{i}\left(d\right),\;P\left(H^{i}\left(0\right)=1.3\right)=1,\;d\in \left[0;D_{0}\right],$$ (4)

the 4-parameters Gompertz type:

$$dH^{i}\left(d\right)=\left[\left(\alpha +\phi_{i}\right)\left(H^{i}(d)-\gamma \right)-\beta \left(H^{i}(d)-\gamma \right)\ln(H^{i}(d)-\gamma )\right]\cdot dd+\sigma \left(H^{i}(d)-\gamma \right)\cdot dW^{i}\left(d\right),\;P\left(H^{i}\left(0\right)=1.3\right)=1,\;d\in \left[0;D_{0}\right],$$ (5)

the Bertalanffy type:

$$dH^{i}\left(x\right)=\frac{\left(\alpha +\phi_{i}\right)\beta \gamma }{e^{\beta d}-\gamma }H^{i}(d)\cdot dd+\sigma H^{i}(d)\cdot dW^{i}\left(d\right),\;P\left(H^{i}\left(0\right)=1.3\right)=1,\;d\in \left[0;D_{0}\right],$$ (6)

the Gamma type:

$$dH^{i}\left(x\right)=\left(\frac{\alpha +\phi_{i}}{d}-\beta \right)H^{i}(d)\cdot dd+\sigma H^{i}(d)\cdot dW^{i}\left(d\right),\;P\left(H^{i}\left(0.001\right)=1.3\right)=1,\;d\in \left[0.001;D_{0}\right],$$ (7)

where *W*^*i*^(*d*), *d*≥0 are the independent standard Brownian motions, *W*^*i*^(*d*) and *ϕ*_*j*_ are mutually independent for all 1*≤i*,*j≤M*, and M is the total number of plots used for model fitting. The term *P*(*H*^*i*^(0) = 1.3) = 1 or *P*(*H*^*i*^(0.001) = 1.3) = 1 (for Eq 7) ensures that if d = 0, then H^i^ = 1.3.

Taking into account the analytical expressions of the deterministic term and the stochastic term specified by Eqs 3–7, both terms fulfil the Lipschitz restriction on growth conditions for the existence and unicity of the solutions of the SDEs defined by Eqs 3–7 [18]. Transforming Eq 3 by *Y*^*i*^(*d*) = *e*^*βd*^*H*^*i*^(*d*), Eqs 4, 6 and 7 by *Y*^*i*^(*d*) = *e*^*βd*^ln(*H*^*i*^(*d*)), and Eq 5 by *Y*^*i*^(*d*) = *e*^*βd*^ln(*H*^*i*^(*d*)−*γ*), and applying Ito's [17] formula, we deduce that the solution, *H*^*i*^(*d*), of Eq 3 has a normal distribution $N\left(\mu_{V}(d),\lambda_{V}^{2}(d)\right)$ and the solutions, *H*^*i*^(*d*), of Eqs 4–7 have lognormal distributions, respectively, $N\left(\mu_{G3V}(d),\lambda_{G3}^{2}(d)\right)$, $N\left(\mu_{G4}(d),\lambda_{G4}^{2}(d)\right)$, $N\left(\mu_{B}(d),\lambda_{B}^{2}(d)\right)$, and $N\left(\mu_{G}(d),\lambda_{G}^{2}(d)\right)$. The conditional probability density, mean, and variance functions were deduced in Appendix B.

An approximated maximum likelihood procedure (see Appendix C) was used for the estimation of the fixed effects parameters and random effects by assuming that tree height and diameter observations are without measurement noise.

### Data

The data used for developing the models were obtained from the Lithuanian National Forest Inventory (LNFI) (2006–2010). The NFI plots are systematically distributed using a grid of 4x4 km squares with a random starting point. The sample plots are arranged into triangle distributed clusters with a distance between angles of 2 km. Each cluster has 4 sample plots. They are situated on each 250 m length side of square 25 m from its angles [19]. At plot establishment, the following data were recorded for every sample tree: the species, the diameter over bark at 1.30 m high and measured to the nearest millimeter and the total height to the nearest quarter meter. The tree diameters were measured with outside calipers in two perpendicular directions. A total of 3,455 plots (500 m^2^) of Scots pine trees were chosen from the LNFI 2006–2010 database. A random sample of 1,999 plots (7,343 trees) was selected for model estimation, and the remaining dataset of 1,456 plots (5,413 trees) was utilized for model validation. Only measurements from live trees without top damage were included in the statistical analysis. Summary statistics for the diameter at breast height (d), the total height (h) and the age (A) for all of the trees used in model estimation and validation datasets are presented in Table 1. Table 2 presents the distribution of the number of trees per plot measurements from both datasets. It should be noted that data on the number of plots with greater than 10 measured trees were very limited.

**Table 1:** Datasets summary statistics.

Data	Number of trees	Min	Max	Mean	St. Dev.	Number of trees	Min	Max	Mean	St. Dev.
Estimation						Validation				
**d (cm)**	7343	15.10	66.10	27.33	8.69	5413	15.10	74.30	27.29	8.68
**h (m)**	7343	6.70	37.80	22.10	4.44	5413	7.10	37.80	21.98	4.39
**A (yr)**	7343	17	221	65.90	23.62	5413	17	192	66.75	23.85

**Table 2:** Distribution of the number of trees per plot measurement.

Number of trees per plot	1	2	3	4	5	6	7	8	9	10	11	12	13	14
**Estimation dataset**														
**Number of plots**	125	492	407	394	293	146	81	37	15	5	2	1	0	1
**Validation dataset**														
**Number of plots**	76	362	295	282	219	111	72	26	7	3	2	1	0	0

## Results

To examine the impact of fixed and random effects on the prediction of the height distribution, the maximum likelihood estimators (Eqs A.2 and A.10) were calculated using the NLPSolve procedure in MAPLE 11 [20]. The models with fixed effects and mixed effects were evaluated based on Akaike’s [21] information criterion (AIC), which was defined as follows:

$$AIC=-2\cdot LL_{K}^{s}+2\cdot p,\;s=1,2,\;K\in \left\{V,G3,G4,B,G\right\},$$ (8)

where $LL_{K}^{s}$ is the log-likelihood function and *p* is the number of parameters in the model. The nested models with the smallest AIC value are considered to be the best. Using the estimation dataset presented **i**n Table 1, the parameter estimates of the fixed effects and mixed effects SDEs height-diameter models, defined by Eqs 3–7, are summarized in Table 3. The standard errors of the parameter estimates were calculated by Eq C12. All of the parameters of the fixed effects and mixed effects SDEs height-diameter models are highly significant (*p* < 0.001). The AIC values for the fixed effects SDEs height-diameter models were more than for mixed effects models, indicating that random effects are needed in the height-diameter SDEs.

**Table 3:** Estimated parameters and AIC for all height-diameter models applied to the estimation dataset.

Models		Parameters				
	α	β	γ	σ	σ_ϕ_	AIC
**Fixed effects**						
**Gamma**	0.2557 (0.0007)	-0.0083 (0.0003)	-	0.0340 (0.0003)	-	35177.793
**Vasicek**	30.6854 (0.0026)	0.0479 (8.7*10^−6^)		1.0568 (0.0001)	-	38138.604
**Gompertz 4-parameters**	0.2825 (0.0028)	0.0502 (0.0004)	-245.9571 (16.3678)	0.0040 (0.0003)	-	38184.947
**Gompertz 3-parameters**	0.3721 (0.0023)	0.1128 (0.0008)	-	0.0786 (0.0007)	-	39515.975
**Bertalanffy**	0.9748 (0.0926)	0.0436 (0.0052)	0.9625 (0.0128)	0.0335 (0.0003)	-	40072.053
**Mixed effects**						
**Gamma**	0.2663 (0.0004)	-0.0034 (0.0001)	-	0.0165 (0.0002)	0.0164 (0.0003)	29773.814
**Vasicek**	26.6348 (0.0739)	0.0667 (0.0005)	-	0.6059 (0.0448)	3.9371 (0.6891)	33262.853
**Gompertz 4-parameters**	0.3682 (0.0043)	0.0710 (0.0007)	-152.6744 (2.4079)	0.0036 (0.0001)	0.0016 (3.6*10^−5^)	33343.351
**Gompertz 3-parameters**	0.4324 (0.0019)	0.1354 (0.0007)	-	0.0429 (0.0006)	0.0238 (0.0005)	34733.167
**Bertalanffy**	0.6770 (0.0049)	0.0436 (0.0007)	0.9893 (0.0004)	0.0164 (0.0002)	0.0404 (0.0006)	34785.470

### Height distributions

**Fig 1:**
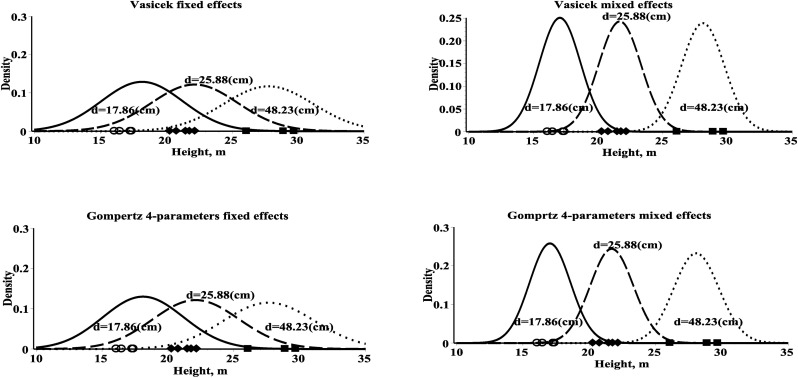
Height’s conditional probability density functions (Eqs B.1, B.4, B.7, B.10 and B.13) for three different plots within estimation dataset. Left–fixed effects models; right–mixed effects models; first plot–solid line and height’s dataset–cross; second plot–dash line and height’s dataset–diamond; third plot–dot line and height’s dataset–box; mean diameter within a plot is recorded in the graph.

Tree height structure is a basic modeling component of many complex forest yield models relating individual tree characteristics with stand variables. The distribution of the tree height, as a diameter dependent variable, can be approximated by classifying diameter and applying the desired transformation to the mean tree of the class [22]. A more convenient way to derive tree height distributions for a given diameter is the use of SDEs. This paper described research aimed at deriving tree height probability densities for a given diameter (Eqs B.1, B.4, B.7, B.10 and B.13) by directly fitting the SDEs (Eqs 3–7) to the diameter and height observations. Fig 1 demonstrates the estimated probability density functions of tree height for a given diameter (Eqs B.1, B.4, B.7, B.10 and B.13) for three randomly selected plots from the estimation dataset. These probability density functions indicate that density curves are steeper for the young stands and less steep for the mature stands. On the other hand, Fig 1 shows that the mixed effects probability density functions are characterized as having smaller variances than the fixed effects probability density functions.

Several empirical methods are available for comparing conditional probability densities, as has been illustrated by [23]. In the present paper, a well-known measure of distributional accuracy named by Kullback-Leibler Information Criterion (KLIC) [24] was utilized. We are interested in comparing two conditional probability density functions $f_{A}\left(h,d|{\hat{\theta }}_{s}\right)$ and $f_{B}\left(h,d|{\hat{\theta }}_{t}\right)$, $\hat{\theta }\in \left\{\left(\hat{\theta_{K}^{1}},0\right),\left(\hat{\theta_{K}^{2}},\hat{\phi }\right)\right\}$, 1 < *s*,*t* < 2, *A*,*B* ∈ {*V*,*G*3,*G*4,*B*,*G*}. Therefore, in particular, we choose conditional probability density $f_{A}\left(h,d|{\hat{\theta }}_{s}\right)$ over $f_{B}\left(h,d|{\hat{\theta }}_{t}\right)$ if:

$$KLIC=E\left(\ln\left(f_{A}\left(h,d|{\hat{\theta }}_{s}\right)\right)-\ln\left(f_{B}\left(h,d|{\hat{\theta }}_{t}\right)\right)\right)>0,\;1\leq s,t\leq 2,\;A,B\in \left\{V,G3,G4,B,G\right\}$$ (9)

Under appropriate conditions, the KLIC has limiting distribution under the null, and is consistent against all possible fixed alternatives. The expression for KLIC in Eq 9 depends on the unknown expectation E(⋅). We consider estimating KLIC by a discrete height sample ($h^{i}=h_{1}^{i},\;h_{2}^{i},\ldots ,h_{n_{i}}^{i}$) at diameters ($d^{i}=d_{1}^{i},d_{2}^{i},\ldots ,d_{n_{i}}^{i}$) analogue:

$$KLIC=\frac{1}{n}\sum_{i=1}^{M}\sum_{j=1}^{n_{i}}\ln\left(f_{A}\left(h_{j}^{i},d_{j}^{i}|\hat{\theta_{K}^{s}},\hat{\phi_{i}}\right)\right)-\ln\left(f_{B}\left(h_{j}^{i},d_{j}^{i}|\hat{\theta_{K}^{t}},\hat{\phi_{i}}\right)\right),\;1\leq s,t\leq 2,\;A,B\in \left\{V,G3,G4,B,G\right\},$$ (10)

where *n*_*i*_ is the number of observed trees of the i*th* plot, *i* = 1,2,…,*M*.

Analysis of paired comparison of the five conditional probability densities, described in Section 2 by Eqs B.1, B.4, B.7, B.10 and B.13 was performed by KLIC calculated using the estimation dataset. The results of comparisons are presented in Table 4. As we see in Table 4, the Vasicek type conditional probability density function of the tree height with mixed effects (see Table 4, values above diagonal) and fixed effects (see Table 4, values below diagonal) are superior to the other densities and the worst conditional probability density function is the Gompertz (3-parameters) type with mixed and fixed effects. All mixed effects density functions are superior to corresponding fixed effects parameters density functions (see bold values in diagonal Table 4).

**Table 4:** Comparison of the conditional probability density functions.

Model	Vasicek	Bertalanffy	Gamma	Gompertz 4-parameters	Gompertz 3-parameters
**Vasicek**	**0.8186**	0.1103	0.1180	0.1630	0.2521
**Bertalanffy**	0.1315	**0.8398**	0.0077	0.0527	0.1418
**Gamma**	0.1450	0.0134	**0.8455**	0.0449	0.1341
**Gompertz 4-parameters**	0.0996	-0.0319	-0.0453	**0.7552**	0.0892
**Gompertz 3-parameters**	0.1904	0.0588	0.0454	0.0908	**0.7568**

### Height-diameter models

**Fig 2:**
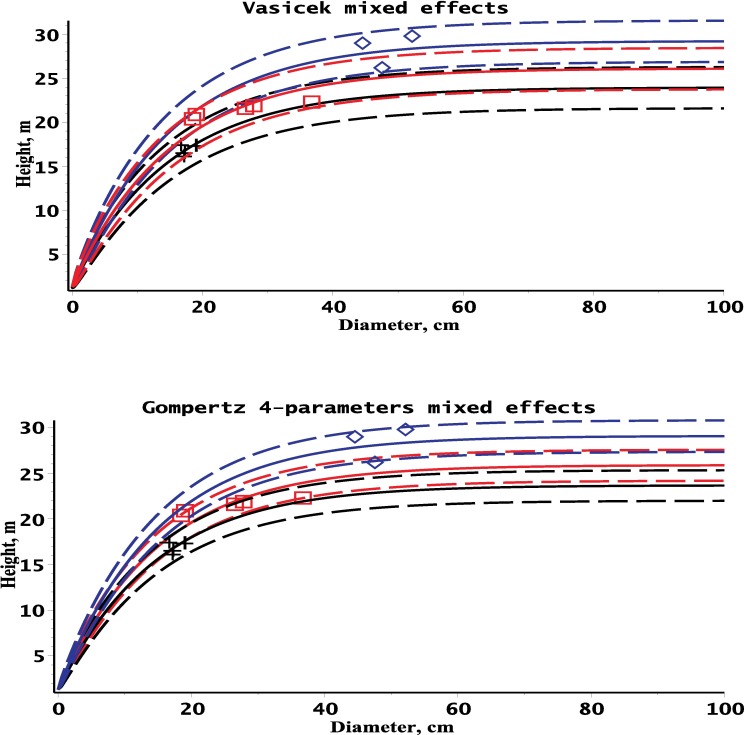
Mean (Eqs B.16 and B.20) and standard deviation (Eqs B.17 and B.21) curves of the height for the 3 randomly selected plots within estimation dataset. First plot–black color, diameter and height dataset–cross; second plot–blue color, diameter and height dataset–diamond; third plot–red color, diameter and height dataset–box; mean height curve—solid line; mean ± standard deviation curve–dash line.

Many comparisons between the different models or ecoregions have been carried out to identify the appropriate height–diameter relationships within stands. The height dynamics defined by Eqs B.16, B.18, B.20, B.22 and B.24 are affected by many processes and vary among stands. Fig 2 illustrates the influence of the plot within Lithuanian pine forests on the mean and standard deviation of height-diameter dynamics using the Vasicek and 4-parameters Gompertz diffusion processes and random-effects parameter, *ϕ*, for the 3 randomly selected plots from the estimation dataset. The parameter estimates for each plot are calculated by adding the fixed effect parameter and random effect. Therefore, considering the asymptotic maximum height parameter, α+ϕ, for the Vasicek type model, the values varied from plot to plot. Fig 2 shows significant differences of tree height dynamics among the sample plots.

To understand the advantages of the height-diameter equations (Eqs B.16, B.18, B.20, B.22 and B.24), fixed effects models, mixed effects models and mixed effects models with the random effects set to zero scenarios were used to predict tree height in both the estimation and validation datasets. The performance statistics of new developed tree height’s equations included three statistical indices: prediction accuracy, *δ*, which combines the mean bias, B, and the variation, *ξ*, of the biases, enabling improved assessment of model accuracy; an adjusted coefficient of determination, ${\bar{R}}^{2}$, which reflects the part of the total variance explained by the equation; and Akaike’s information criterion, AICC, which measure the quality of the height-diameter equation for a given dataset. The expressions for these statistics are as follows:

$$\delta =\sqrt{B^{2}+\xi },\;(B=\frac{1}{n}\sum_{i=1}^{n}\left(y_{i}-\hat{y_{i}}\right),\;\xi =\frac{1}{n-1}\sum_{i=1}^{n}{\left(y_{i}-\hat{y_{i}}-B\right)}^{2})$$

$${\bar{R}}^{2}=1-\frac{n-1}{n-p}\frac{\sum_{i=1}^{n}{\left(y_{i}-\hat{y_{i}}\right)}^{2}}{\sum_{i=1}^{n}{\left(y_{i}-\bar{y}\right)}^{2}}$$

$$AICC=n\ln(RSS)+2p\;(RSS=\sum_{i=1}^{n}{\left(y_{i}-\hat{y_{i}}\right)}^{2})$$

where *n* is the total number of observations used to estimate the height-diameter model, p is the number of model parameters, and *y*_*i*_, $\hat{y_{i}}$, and $\bar{y}$ are the measured, predicted, and average values of the dependent variable (total tree height), respectively.

Table 5 presents the performance statistics for the tree height’s models for all three scenarios; these include the fixed effects model, the mixed effects model and the mixed effects model with random effects set to zero, illustrating the extent to which the inclusion of the random effects improved the performance statistics for both the estimation and validation datasets. The random effects for the validation dataset were calibrated using Eqs D.1–D.5, respectively. The results of this study show (see Table 5, Akaike’s information criterion) that the SDEs Vasicek and Gompertz 4-parameters type tree height’s models with mixed effects are significantly superior at predicting tree height compared to the other newly developed models. Compared to the basic fixed effects models, the mixed effects models show better performance with lower bias and prediction accuracy, and with a higher adjusted coefficient of determination evaluated over the entire dataset. The mixed effects models with random effects set to zero show the worst performance, with greater bias and prediction accuracy, and with a lower adjusted coefficient of determination evaluated over the entire dataset. The fixed effects models, the mixed effects models, and the mixed effects models with random effects set to zero have very similar fit statistics for both the estimation and validation datasets.

**Table 5:** Fit statistics for all of the scenarios used*.

Models	Fixed effects			Mixed effects			Mixed effects, *ϕ* = 0		
	*δ*	${\bar{R}}^{2}$	AICC	*δ*	${\bar{R}}^{2}$	AICC	*δ*	${\bar{R}}^{2}$	AICC
	**Estimation dataset**								
**Vasicek**	3.2576	0.4621	82712.75	1.4025	0.9003	70338.84	3.3667	0.4270	83177.48
**Gompertz 4-parameters**	3.2574	0.4621	82714.09	1.4021	0.9003	70335.78	3.3753	0.4243	83212.99
**Bertalanffy**	3.2640	0.4603	82738.25	1.4478	0.8937	70807.43	3.3899	0.4317	83118.15
**Gompertz 3-parameters**	3.3019	0.4474	82910.37	1.4484	0.8936	70811.32.	3.4663	0.3911	83623.51
**Gamma**	3.4016	0.4142	83339.16	1.4832	0.8885	71159.34	3.3969	0.4303	83135.02
**Models**	**Validation dataset**								
**Vasicek**	3.1937	0.4720	59103.94	1.4140	0.8964	50290.13	3.2970	0.4369	59453.04
**Gompertz 4-parameters**	3.1934	0.4721	59104.84	1.4143	0.8963	50294.93	3.3051	0.4342	59479.82
**Bertalanffy**	3.2035	0.4699	59125.96	1.4272	0.8945	50390.49	3.3114	0.4411	59413.85
**Gompertz 3 parameters**	3.2349	0.4586	59239.11	1.4560	0.8902	50604.51	3.4149	0.4015	59783.72
**Gamma**	3.3540	0.4196	59916.50	1.4719	0.8878	50722.98	3.3234	0.4379	59444.05

* Height-diameter models are ranked with regard to their AICC for the mixed effect scenarios and validation dataset.

**Fig 3:**
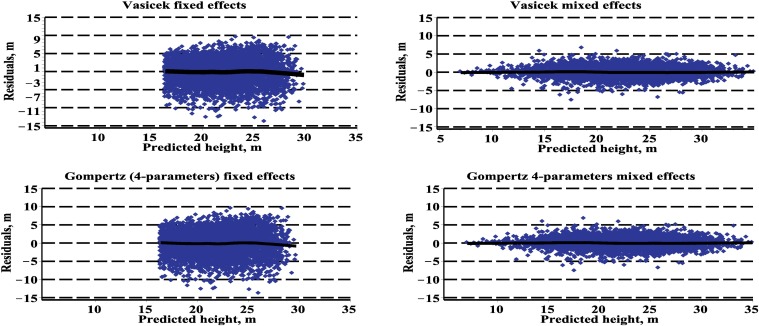
Residuals and the lowess curve of the tree height fixed effects and mixed effects models for the validation dataset.

The plots of the residuals versus predicted heights and the lowess line [25], estimated for the validation dataset, in the fixed effects and mixed effects scenarios (random effects for the validation dataset were calibrated by Eqs D.1–D.5) are presented in Fig 3. Fig 3 shows that the residuals that were calculated using the mixed effects scenario are distributed more symmetrically around zero, with approximately constant variance, compared with the fixed effects scenario. A non-parametric smoothing line, called a lowess line, shows a clear trend in the middle range of predicted height; however, what happens at the extremes is dictated by relatively little data.

### Quantile regression

The conditional tree height’s mean, defined by Eqs B.16, B.18, B.20, B.22 and B.24, illuminates just one aspect of the conditional distribution of a tree height and yet neglects all other features of possible interest. Quantile regression model allows the predictor variable to have a more complex relationship with the response variable [26]. Our developed tree height’s conditional probability density functions for a given diameter (Eqs B.1, B.4, B.7, B.10 and B.13) enables us to write the quantile equation of the tree height to any desired conditional quantile of the height’s distribution. Forest researchers are not mainly interested in quantifying the conditional central tendency of the tree height. Evidently, quantile tree height models also allow us to explore the lower boundary relationship which covers cramped trees with a very slender trunk. On the other hand, the exact conditional density functions, defined by Eqs B.1, B.4, B.7, B.10 and B.13, can be employed in practice by the quantile regression, which allow us to make height predictions using intervals that contain the tree height for a given diameter, with a specific probability, 0<*p<1*. For the Vasicek type model the quantiles functions are defined as follows:

$$Q_{V}(d,p)=\inf\left\{y:\Phi \left({\hat{\mu }}_{V}(d,\hat{\phi });\hat{\lambda_{V}^{2}}(d)\right)\geq p\right\}=\Phi_{p}^{-1}\left({\hat{\mu }}_{V}(d,\hat{\phi });\hat{\lambda_{V}^{2}}(d)\right)$$

and for the Gompertz 4-parameters type model:

$$Q_{G4}(d,p)=\inf\left\{y:LN\left({\hat{\mu }}_{G4}(d,\hat{\phi });\hat{\lambda_{G4}^{2}}(d)\right)\geq p\right\}=\hat{\gamma }+LN_{p}^{-1}\left({\hat{\mu }}_{G4}(d,\hat{\phi });\hat{\lambda_{G4}^{2}}(d)\right).$$

**Fig 4:**
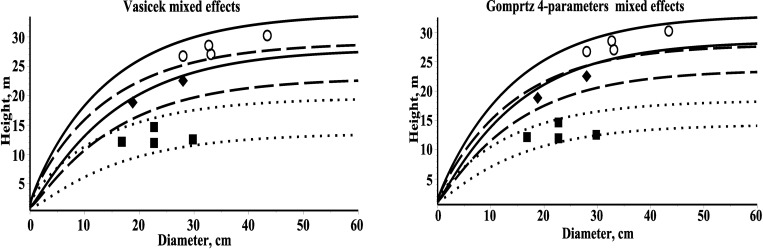
10% and 90% quantile functions for the mixed-effects models and three different plots within estimation dataset. First plot–solid line and dataset (diameter and height)–circle; second plot–dash line and dataset (diameter and height)–diamond; third plot–dot line and dataset (diameter and height)–box.

For example, the 10% quantile function, $\hat{h}{\left(d\right)}_{0,1}=Q(d,0.1)$, (splits off the lowest 10% tree height predictions from the highest 90%) and the 90% quartile function, $\hat{h}{\left(d\right)}_{0.9}=Q(d,0.9)$, (splits off the highest 10% of tree height predictions from the lowest 90%). For three randomly selected plots 10% and 90% quantile functions are presented in Fig 4.

### Slenderness ratio

Tree height to diameter ratio (slenderness ratio) is regarded as an index of the resistance of trees to windthrow and competition for light, and its mean value may be useful in determining stand stability. The slenderness ratio is calculated by dividing the tree height to its diameter at breast height. For the fixed effects and mixed effects SDEs height-diameter models, defined by Eqs 3–7, the slenderness functions are defined as follows:

$$R_{K}(d)=\int_{0}^{50}\frac{h}{d}f_{K}\left(h,d|\hat{\theta }\right)\cdot dh,\;\hat{\theta }\in \left\{\left(\hat{\theta_{K}^{1}},0\right),\left(\hat{\theta_{K}^{2}},\hat{\phi }\right)\right\},\;K\in \left\{V,G3,G4,B,G\right\}.$$

**Fig 5:**
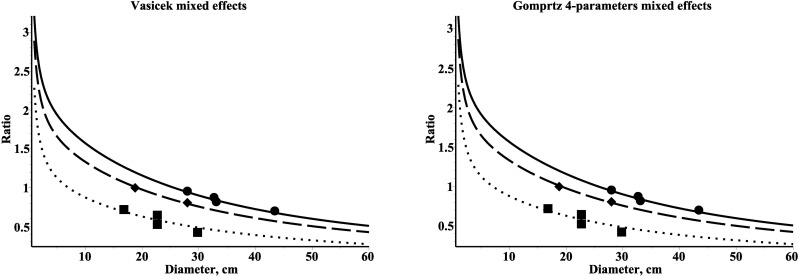
Slenderness dynamics for mixed-effects models and three different plots within estimation dataset. First plot described by solid line–dataset (height/diameter) by circle; second plot described by dash line–dataset (height/diameter) by diamond; third plot described by dot line–dataset (height/diameter) by box.

The slenderness ratio dynamics for three randomly selected plots are presented in Fig 5.

The findings of our investigation generally support that the height’s conditional probability densities driven by diameter correctly predict slenderness ratio (see Fig 5). Furthermore, all new developed tree height’s distribution models show a decrease of slenderness ratio with increasing diameter.

### Mean stem volume

The fixed effects and mixed effects height’s conditional probability density functions allow us to revise mean stem volume calculation in the following form:

$$\bar{V}(d)=\int_{h>0}V(d,h)\cdot f_{K}\left(h,d|\hat{\theta }\right)\cdot dh,\;\hat{\theta }\in \left\{\left(\hat{\theta_{K}^{1}},0\right),\left(\hat{\theta_{K}^{2}},\hat{\phi }\right)\right\},\;K\in \left\{V,G3,G4,B,G\right\}.$$

**Fig 6:**
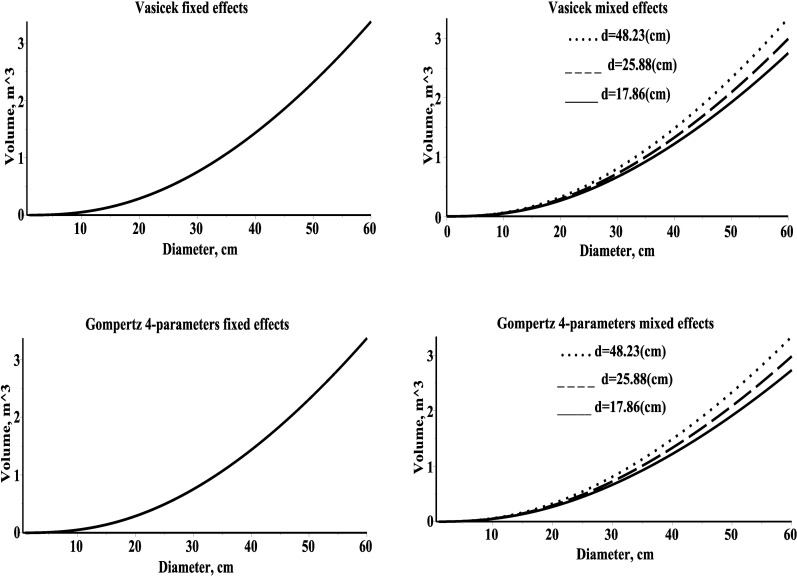
Mean stem volume for three different plots within estimation dataset. Left–fixed effects models; right–mixed effects models; first plot (mean diameter 48.23)–solid line; second plot (mean diameter 26.88)–dash line; third plot (mean diameter 17.86)–dot line; mean diameter within a plot is recorded in graph.

Here *V*(*d*,*h*) is the stem volume regression function of power form [27], $V=\beta_{1}d^{\beta_{2}}h^{\beta_{3}}$, where parameters β_1_, β_2_, and β_3_ are to be estimated. The selection of stem volume model was basically motivated by the available measured tree level characteristics. Parameter estimates were calculated by weighted least squares technique. The estimators and their standard deviations (in parenthesis) are, ${\hat{\beta }}_{1}=5.8*10^{\text{-}5}\;(5.8*10^{\text{-}6})$, ${\hat{\beta }}_{2}=1.8801\;(0.028)$, ${\hat{\beta }}_{3}=0.9723\;(0.045)$ [12]. The relationship between the mean stem volume and the diameter of a tree for the fixed effects and mixed effects Vasicek and Gompertz 4-parameters type models are shown in Fig 6.

The direct effects of stand variables such as site index and management practices and thinning could be included in the new developed models (see [28], [29]); however, their indirect effect via mixed effects (see right side Fig 6) has been included in mixed effects tree mean volume models.

### Coefficient of variation for height and volume

The coefficient of variation is typically used to indicate the precision of the dispersion of datasets and is also often used to compare numerical distributions measured at different scales. Tree height based and tree volume based quantifications of the stand structural diversity can be performed using the coefficient of variation. The coefficient of variation reaches its maximum with two-storied stands. The coefficient of variation of tree height (tree volume) measures the variability of tree height (tree volume) relative to its mean and relates the mean and standard deviation by expressing the standard deviation as a percentage of the mean. To further discuss the results of this study, the coefficient of variation, which may help examine dispersion in tree heights occurring at diameter d, is defined by:

$$CV_{K}^{h}\left(d\right)=\frac{\sqrt{v_{K}(d)}}{m_{K}(d)}\cdot 100,\;K\in \left\{V,G3,G4,B,G\right\}$$

and dispersion in tree volumes occurring at diameter d:

$$CV_{K}^{v}\left(d\right)=\frac{\sqrt{\int_{-\infty }^{+\infty }V{\left(d,h\right)}^{2}\cdot f\left(h,d|\hat{\theta }\right)\cdot dh-\bar{V}{\left(d\right)}^{2}}}{\bar{V}(d)}\cdot 100,\;\hat{\theta }\in \left\{\left(\hat{\theta_{K}^{1}},0\right),\left(\hat{\theta_{K}^{2}},\hat{\phi }\right)\right\},\;K\in \left\{V,G3,G4,B,G\right\}.$$

**Fig 7:**
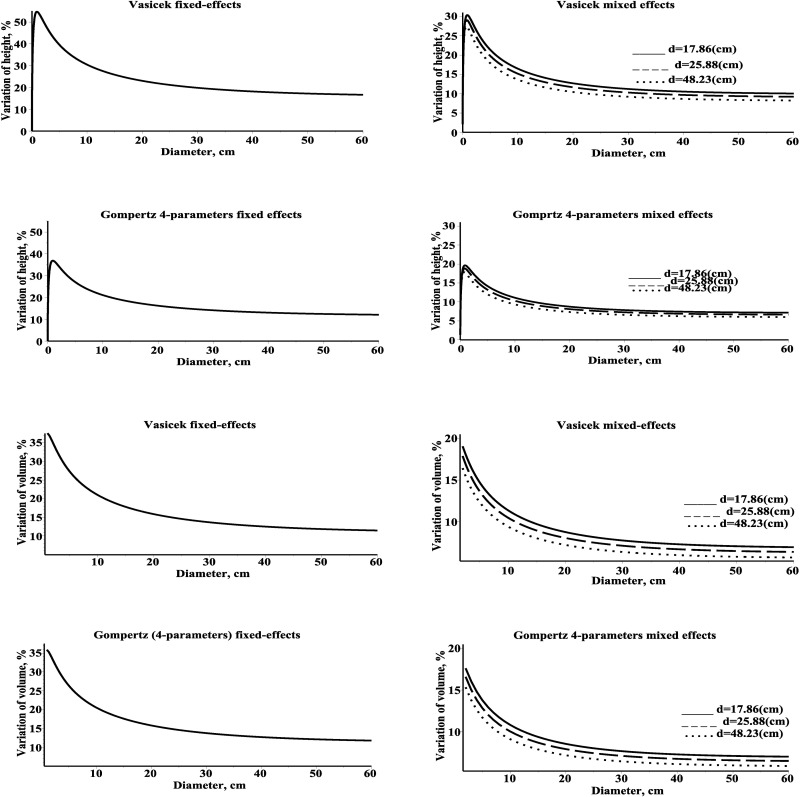
Coefficient of variation of tree height and volume for three different plots within estimation dataset. Left–fixed effects; right–mixed effects models; first plot (mean diameter 17.86)–solid line; second plot (mean diameter 25.88)–dash line; third plot (mean diameter 48.23)–dot line; mean diameter within a plot is recorded in graph.

Fig 7 shows a plot of the coefficient of variation as a function of a diameter using the mean trend and standard deviation functions. In both cases (height and volume), the coefficient of variation of the tree height and volume evolves into a stationary coefficient of variation. The coefficient of variation based on tree height (volume) decreases with an increase in diameter.

## Discussion

The models commonly used of height distribution fitting in a forest stand are supplemented by tree height’s measurements. However, in the Lithuanian National Forest Inventory foresters measure no more than 15 heights (see Table 2) of pine trees per stand. For estimating the parameters by traditionally used maximum likelihood technique such sample sizes are too small [5], [30]. New developed height distribution models based on mixed effects parameters diffusion processes overcome such weakness. The pioneer of the SDE approach in forest growth modeling is Suzuki [31]. In this paper for height-diameter evolution were used linear and non-linear SDEs from the Ornstein—Uhlenbeck family by incorporating random effects into deterministic (drift) term. This extended model describes the within-stand variation in data through the system noise reflecting the random fluctuations around the corresponding theoretical height-diameter curve and the between-stand variation in data through the random effects. The maximum likelihood estimation procedure converged for all five diffusion processes using the estimated dataset from LNFI.

In order to predict the parameters of the tree size probability density function for a new stand, traditionally were carried out regression models from the different stand variables [32]. If the diameter and height of a sub-sample of trees are known, then for new developed height distributions based on univariate diffusion processes the random effects can be calibrated by Eqs D.1–D.5.

Quantifying variability in tree height at a given diameter by a distribution law has both theoretical and practical value. First, knowledge of the tree height distribution in forest stands is important for understanding of competition and self-thinning which must studied not in the mean size of trees but in the size structure of trees in a forest stand. Second, understanding tree height distribution at a given diameter is important for improving estimates of stand biomass and carbon storage. To describe how tree height distribution vary across regional scales, we developed new empirical distributions of tree height at a given diameter across the Scots pine trees in Lithuania. In this paper our specific objectives were to test (1) what new developed probability density function based on stochastic differential equation height-diameter evolution provides the best fit, (2) how new developed models explain observed variation in the probability density functions, the mean height-diameters, quantile height-diameter, mean slenderness ratio and mean stem volume relationships across the Scots pine trees in Lithuania, and (3) how to describe the mean height-diameter and mean stem volume relationships fit in terms of the relative sizes defined by coefficient of variation.

The Kullback-Leibler Information Criterion [24] was used to compare all new developed conditional probability density functions using the estimation dataset. The conditional probability density function derived from the Vasicek type height-diameter univariate diffusion process showed better results than the other used stochastic processes (Table 4). All mixed effects parameters probability density functions are superior to corresponding fixed effects parameters density functions (see bold values in diagonal, Table 4). Theoretical validating that a height dataset observed at discrete diameters follows univariate probability density functions defined by Eqs B.1, B.4, B.7, B.10 and B.13 is not easy and there is no simple statistical test. The goodness of fit of the estimated univariate density functions (Eqs B.1, B.4, B.7, B.10 and B.13) graphically were illustrated in Fig 1 using fixed effects and mixed effects parameters scenarios and three randomly selected plots from an estimation dataset by plotting the estimated probability density functions and height’s measurements. Fig 1 showed that the mixed effects and fixed effects parameters estimated probability density functions well capture the main features of the data from three randomly selected plots. The height-diameter evolution can be written using a wide range of mathematical relationships from linearized fixed effects regression equations to nonlinear mixed effects generalized relationships. Mathematical technique of a system of uniform diameter and height regional functions is the approach known as the generalized model. The mixed effects regression models are able to achieve the same results than the generalized model [10, 33]. In this study new developed mixed effects parameters height-diameter relationships demonstrated similar statistical indexes as in the nonlinear generalized height-diameter regression models presented by Petrauskas et al. [34].

**Fig 8:**
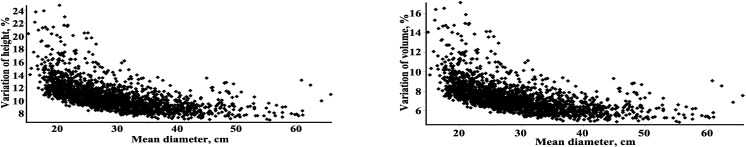
Coefficient of variation of tree height and volume for plots within estimation dataset. Left–tree height; right–stem volume.

In addition, one of the advantages of using diffusion processes for quantifying tree height distribution is that it allows to derive the first two moments about height’s and volume’s evolutions through diameter and to calculate the relative standard deviation (coefficient of variation) for the height and volume. Fig 8 shows the variation of the coefficient of variation in pine trees forest stands from the estimation dataset from LNFI as a function of mean plot diameter using the mixed effects Vasicek type diffusion process. There is an exponential increase of the coefficient of variation as the mean diameter per plot decreases; the coefficients of variation for tree height varies from 6.94% to 24.72% and for stem volume varies from 4.77% to 17.05%.

## Conclusions

This study demonstrated the use of SDEs to quantify tree height distribution at a given diameter in a forest stand using the Lithuanian National Forest Inventory dataset. The results indicated that it is possible to measure mean tree height and volume evolution with an acceptable accuracy over a broad area of Lithuania. Overall, the models explained over 90% of the variation in height predictions observed in the LNFI (2006–2010) dataset. The remaining variation was likely to have resulted from stand variables. Better performance can be expected by introducing stand variables [29]. The diffusion processes based SDE models described here implicitly model spatial effects. The technique we described can be used for developing a new generation of forest growth models.

A system of bivariate stochastic differential equations with mixed-effects parameters could be used to develop tree diameter and height at a given age (or trivariate: diameter, height and stand density at a given age) distribution model. This extension to multivariate SDEs come with an increased computational burden.

Results for both tree height and volume predictions using the mixed effects SDE Vasicek type height-diameter model indicate that the coefficient of variation over all plots for the tree height and volume (at the mean diameter of a plot) takes values from the interval 6.9%–24.8% and 1.7%–16.0%, respectively, and evolves to a stationary value from the interval 6.6%–19.8% and 1.7%–13.0%, respectively.

The field of SDEs is a large and growing area of applied mathematics that is being increasingly used to model biological systems. In this paper, new mixed effects height’s probability density functions for a given diameter were developed using an Ornstein-Uhlenbeck SDE family. Unfortunately, measurements from at least one tree in a stand, or their measure of central tendency (mean, median, mode of diameter and height) are required for the practical calibration of the random effects for a new stand. The use of the mixed effects model enables us to develop a simple model structure without including additional predictor stand variables.

The results showed that the mixed effects Vasicek type tree heights distribution models are superior to the other new developed models.

The variance functions developed here can be applied to generate weights in every linear and nonlinear least squares regression height-diameter model by the weighted least squares form.

## Appendix A

### Deterministic models

The mathematical representation of Mitscherlich growth [35] is derived from physical chemistry, where it describes a first order irreversible chemical reaction. The deterministic height-diameter model used to describe the individual growth of a tree in terms of its size (height), h(d), at instant (diameter), d, can be written in the form of an autonomous differential equation given by the following:

$$\frac{dh\left(d\right)}{dd}=\beta \left(\alpha -h(d)\right),\;h\left(0\right)=1.3,\;d\in \left[0;D_{0}\right],$$ (A.1)

where *D*_0_ is the upper limit on the diameter at the breast height. Height dynamics are irreversible, and the growth rate is proportional to the difference between the asymptotic maximum height, α, and the already formed tree height, h(d), β is the proportionality constant (β>0). The formula describing a Mitscherlich type height-diameter trajectory takes the form:

$$h(d)=\alpha +\left(1.3-\alpha \right)\exp(-\beta d),\;d\in \left[0;D_{0}\right].$$ (A.2)

The changes in tree height, h(d), using deterministic ordinary differential equations, developed by Gompertz [36], for 2-parameters and 3-parameters models, respectively, are described as follows:

$$\frac{dh(d)}{dd}=\alpha h(d)-\beta h(d)\ln\left(h(d)\right),\;d\in \left[0;D_{0}\right],$$ (A.3)

$$\frac{dh(d)}{dd}=\alpha \left(h(d)-\gamma \right)-\beta \left(h(d)-\gamma \right)\ln\left(h(d)-\gamma \right),\;d\in \left[0;D_{0}\right].$$ (A.4)

The formulas describing a Gompertz type height-diameter trajectory for 2-parameters and 3-parameters models, respectively, are as follows:

$$h(d)=\exp\left(\frac{\alpha }{\beta }-\left(\frac{\alpha }{\beta }-\ln(1.3)\right)\cdot \exp\left(-\beta d\right)\right),\;d\in \left[0;D_{0}\right],$$ (A.5)

$$h(d)=\gamma +\exp\left(\frac{\alpha }{\beta }-\left(\frac{\alpha }{\beta }-\ln(1.3-\gamma )\right)\cdot \exp\left(-\beta d\right)\right),\;d\in \left[0;D_{0}\right].$$ (A.6)

where α is the intrinsic growth rate of the height, β is the growth deceleration factor, γ is a threshold parameter, and $\exp\left(\frac{\alpha }{\beta }\right)$ represents the largest height size that the tree can sustain.

Von Bertalanffy (for a review, see example in Román-Román et al. [13]) hypothesized that the growth of an organism could be represented as the difference between the synthesis and degradation of its building materials. There are few theoretical equations formulated specifically for biology applications. In this paper, the tree height, h(d), are described using an ordinary differential equation:

$$\frac{dh(d)}{dd}=\frac{\alpha \beta \gamma }{e^{\beta d}-\gamma }h(d),\;d\in \left[0;D_{0}\right].$$ (A.7)

where α, β, and γ are unknown fixed effects parameters. The formula describing the Bertalanffy trajectory follows the form of a sigmoidal function:

$$h(d)=1.3{\left(\frac{1-\gamma e^{-\beta d}}{1-\gamma }\right)}^{\alpha },\;d\in \left[0;D_{0}\right].$$ (A.8)

The changes in tree height, h(d), using the well-known regulated Malthusian growth process [37], are described in the following form:

$$\frac{dh(d)}{dd}=\left(\frac{\alpha }{d}-\beta \right)h(d),\;d\in \left[0.001;D_{0}\right].$$ (A.9)

The formula describing the Gamma (Malthusian) trajectory follows the form:

$$h(d)=1.3{\left(\frac{d}{0.001}\right)}^{\alpha }e^{-\beta \left(d-0.001\right)},\;d\in \left[0.001;D_{0}\right].$$ (A.10)

## Appendix B

### Conditional probability densities

The solution, *H*^*i*^(*d*), of Eq 3 has a normal distribution $N\left(\mu_{V}(d),\lambda_{V}^{2}(d)\right)$ with conditional probability density, mean, and variance, respectively [29]:

$$f_{V}\left(h,d|\alpha ,\beta ,\sigma ,\phi_{i}\right)=\sqrt{\frac{1}{2\pi \lambda_{V}^{2}(d)}}\exp\left(-\frac{{\left(h-\mu_{V}(d)\right)}^{2}}{2\lambda_{V}^{2}(d)}\right),$$ (B.1)

$$\mu_{V}(d)=1.3e^{-\beta d}+\frac{\alpha +\phi_{i}}{\beta }\left(1-e^{-\beta d}\right),$$ (B.2)

$$\lambda_{V}^{2}(d)=\frac{1-e^{-2\beta d}}{2\beta }\sigma_{}^{2},$$ (B.3)

and the solutions, *H*^*i*^(*d*), of Eqs 4–7 have lognormal distributions, with conditional probability density, means, and variance, respectively [28, 38]:

$$f_{G3}\left(h,d|\alpha ,\beta ,\sigma ,\phi_{i}\right)=\frac{1}{\sqrt{2\pi \lambda_{G3}^{2}(d)}h}\exp\left(-\frac{{\left(\ln\left(h\right)-\mu_{G3}(d)\right)}^{2}}{2\lambda_{G3}^{2}(d)}\right),$$ (B.4)

$$\mu_{G3}(d)=\ln(1.3)\cdot e^{-\beta d}+\left(\alpha +\phi_{i}-\frac{\sigma^{2}}{2}\right)\left(\frac{1-e^{-\beta d}}{\beta }\right),$$ (B.5)

$$\lambda_{G3}^{2}(d)=\frac{1-e^{-2\beta d}}{2\beta }\sigma_{}^{2},$$ (B.6)

$$f_{G4}\left(h,d|\alpha ,\beta ,\gamma ,\sigma ,\phi_{i}\right)=\frac{1}{\sqrt{2\pi \lambda_{G4}^{2}(d)}\left(h-\gamma \right)}\exp\left(-\frac{{\left(\ln\left(h-\gamma \right)-\mu_{G4}(d)\right)}^{2}}{2\lambda_{G4}^{2}(d)}\right),$$ (B.7)

$$\mu_{G4}(d)=\gamma +\ln(1.3-\gamma )\cdot e^{-\beta d}+\left(\alpha +\phi_{i}-\frac{\sigma^{2}}{2}\right)\left(\frac{1-e^{-\beta d}}{\beta }\right),$$ (B.8)

$$\lambda_{G4}^{2}(d)=\frac{1-e^{-2\beta d}}{2\beta }\sigma_{}^{2},$$ (B.9)

$$f_{B}\left(h,d|\alpha ,\beta ,\gamma ,\sigma ,\phi_{i}\right)=\frac{1}{\sqrt{2\pi \lambda_{B}^{2}(d)}h}\exp\left(-\frac{{\left(\ln\left(h\right)-\mu_{G3}(d)\right)}^{2}}{2\lambda_{B}^{2}(d)}\right),$$ (B.10)

$$\mu_{B}\left(d\right)=\ln\left(1.3{\left(\frac{\beta \gamma \left(1-\gamma e^{-\beta d}\right)}{1-\gamma }\right)}^{\left(\alpha_{0}+\phi_{i}\right)}\right),$$ (B.11)

$$\lambda_{B}^{2}(d)=\sigma_{}^{2}d,$$ (B.12)

$$f_{G}\left(h,d|\alpha ,\beta ,\gamma ,\sigma ,\phi_{i}\right)=\frac{1}{\sqrt{2\pi \lambda_{G}^{2}(d)}h}\exp\left(-\frac{{\left(\ln\left(h\right)-\mu_{G}(d)\right)}^{2}}{2\lambda_{G}^{2}(d)}\right),$$ (B.13)

$$\mu_{G}(d)=\ln(1.3)+\left(\alpha +\phi_{i}\right)\ln\left(\frac{d}{0.001}\right)-\left(\beta +\frac{\sigma^{2}}{2}\right)\left(d-0.001\right),$$ (B.14)

$$\lambda_{G}^{2}(d)=\sigma_{}^{2}\left(d-0.001\right).$$ (B.15)

The conditional mean, m(d), and variance, v(d), functions of the tree height, H(d), for all the models (Eqs 14–17) are given by the following expressions [28], [29], [38]:

$$m_{V}(d)=E\left(H^{i}\left(d\right)|H^{i}\left(0\right)=1.3\right)=\alpha +\phi_{i}+(1.3-(\alpha +\phi_{i}))e^{-\beta d},$$ (B.16)

$$v_{V}(d)=Var\left(H^{i}\left(d\right)|H^{i}\left(0\right)=1.3\right)=\frac{1-e^{-2\beta d}}{2\beta }\sigma_{}^{2},$$ (B.17)

$$m_{G3}(d)=E\left(H^{i}\left(d\right)|H^{i}\left(0\right)=1.3\right)=\exp\left(\ln(1.3)\cdot e^{-\beta d}+\frac{1-e^{-\beta d}}{\beta }\left(\alpha +\phi_{i}-\frac{\sigma_{{}_{}}^{2}}{2}\right)+\left(\frac{\sigma^{2}}{4\beta }\left(1-e^{-2\beta d}\right)\right)\right),$$ (B.18)

$$\begin{array}{l} v_{G3}(d)=Var\left(H^{i}\left(d\right)|H^{i}\left(0\right)=1.3\right)\\ =\exp\left(2\left(\ln(1.3)\cdot e^{-\beta d}+\frac{1-e^{-\beta d}}{\beta }\left(\alpha +\phi_{i}-\frac{\sigma_{{}_{}}^{2}}{2}\right)\right)+\frac{\sigma^{2}}{2\beta }\left(1-e^{-2\beta d}\right)\right){\left(\exp\left(\frac{\sigma^{2}}{2\beta }\left(1-e^{-2\beta d}\right)\right)-1\right)}_{}^{2} \end{array},$$ (B.19)

$$m_{G4}(d)=E\left(H^{i}\left(d\right)|H^{i}\left(0\right)=1.3\right)=\gamma +\exp\left(\ln(1.3-\gamma )\cdot e^{-\beta d}+\frac{1-e^{-\beta d}}{\beta }\left(\alpha +\phi_{i}-\frac{\sigma_{{}_{}}^{2}}{2}\right)+\left(\frac{\sigma^{2}}{4\beta }\left(1-e^{-2\beta d}\right)\right)\right),$$ (B.20)

$$\begin{array}{l} v_{G4}(d)=Var\left(H^{i}\left(d\right)|H^{i}\left(0\right)=1.3\right)\\ =\exp\left(2\left(\ln(1.3-\gamma )\cdot e^{-\beta d}+\frac{1-e^{-\beta d}}{\beta }\left(\alpha +\phi_{i}-\frac{\sigma_{{}_{}}^{2}}{2}\right)\right)+\frac{\sigma^{2}}{2\beta }\left(1-e^{-2\beta d}\right)\right)\left(\exp\left(\frac{\sigma^{2}}{2\beta }\left(1-e^{-2\beta d}\right)\right)-1\right) \end{array},$$ (B.21)

$$m_{B}(d)=E\left(H^{i}\left(d\right)|H^{i}\left(0\right)=1.3\right)=1.3{\left(\frac{1-\gamma e^{-\beta d}}{1-\gamma }\right)}^{\left(\alpha +\phi_{i}\right)},$$ (B.22)

$$v_{B}(d)=Var\left(H^{i}\left(d\right)|H^{i}\left(0\right)=1.3\right)={\left(1.3{\left(\frac{1-\gamma e^{-\beta d}}{1-\gamma }\right)}^{\alpha +\phi_{i}}\right)}^{2}\cdot \left(\exp\left(\sigma^{2}d\right)-1\right),$$ (B.23)

$$m_{G}(d)=E\left(H^{i}\left(d\right)|H^{i}\left(0\right)=1.3\right)=1.3{\left(\frac{d}{0.001}\right)}^{\alpha +\phi_{i}}\cdot \exp\left(-\beta \left(d-0.001\right)\right),$$ (B.24)

$$v_{G}(d)=Var\left(H^{i}\left(d\right)|H^{i}\left(0\right)=1.3\right)=1.3^{2}{\left(\frac{d}{0.001}\right)}^{2\left(\alpha +\phi_{i}\right)}\cdot \exp\left(-2\beta \left(d-0.001\right)\right)\cdot \left(\exp\left(\sigma^{2}\left(d-0.001\right)\right)-1\right).$$ (B.25)

## Appendix C

### Maximum likelihood procedure

We consider the SDEs height-diameter models, as defined by Eqs 3–7, from two perspectives. First, the log-likelihood functions are derived for the fixed effects parameters models (in this case the parameters of random effects, *ϕ*_*i*_, *i* = 1,…,*M* are assumed to be equal to its mean value E(*ϕ*_*i*_) = 0). Second, the log-likelihood functions are derived for the mixed effects. In the sequel, *K* ∈ {*V*,*G*3,*G*4,*B*,*G*}, $\theta_{V}^{1}=\theta_{G3}^{1}=\theta_{G}^{1}=\left\{\alpha ,\beta ,\sigma \right\}$, $\theta_{G4}^{1}=\theta_{B}^{1}=\left\{\alpha ,\beta ,\gamma ,\sigma \right\}$ $\theta_{G4}^{2}=\theta_{B}^{2}=\left\{\alpha ,\beta ,\gamma ,\sigma ,\sigma_{\phi }\right\}$, $\theta_{V}^{2}=\theta_{G3}^{2}=\theta_{G}^{2}=\left\{\alpha ,\beta ,\sigma ,\sigma_{\phi }\right\}$, $\theta_{G4}^{2}=\theta_{B}^{2}=\left\{\alpha ,\beta ,\gamma ,\sigma ,\sigma_{\phi }\right\}$.

The fixed effects parameters $\theta_{K}^{1}$, $\theta_{K}^{2}$, *K* ∈ {*V*,*G*3,*G*4,*B*,*G*} are estimated by means of an approximated maximum likelihood procedure using discrete sampling and conditional probability density functions defined by Eqs B.1, B.4, B.7, B.10 and B.13. We assume that all observations are independent (no repeated measurements are used in the dataset for model estimation). Let us consider a discrete height sample ($h_{1}^{i},h_{2}^{i},\ldots ,h_{n_{i}}^{i}$) at diameters ($d_{1}^{i},d_{2}^{i},\ldots ,d_{n_{i}}^{i}$) without measurement errors, where *n*_*i*_ is the number of observed trees of the i*th* plot, *i* = 1,2,…,*M*. The associated likelihood functions for the fixed effects parameters SDEs height-diameter models (the parameters of random effects, *ϕ*_*i*_, *i* = 1,…,*M* are assumed to be equal to the mean value E(*ϕ*_*i*_) = 0), take the following forms:

$$L_{K}^{1}(\theta_{K}^{1})=\prod_{i=1}^{M}\prod_{j=1}^{n_{i}}f_{K}\left(h_{j}^{i},d_{j}^{i}|\theta_{K}^{1},0\right),\;K\in \left\{V,G3,G4,B,G\right\}$$ (C.1)

and the log-likelihood functions are:

$$LL_{K}^{1}(\theta_{K}^{1})=\sum_{i=1}^{M}\sum_{j=1}^{n_{i}}\ln\left(f_{K}\left(h_{j}^{i},d_{j}^{i}|\theta_{K}^{1},0\right)\right),\;K\in \left\{V,G3,G4,B,G\right\},$$ (C.2)

where *n*_*i*_ is the number of observed trees of the i*th* plot *i* = 1,2,…,*M*, $\theta_{K}^{1}$ are fixed effects parameters (the same for all plots), density functions $f_{K}\left(h_{j}^{i},d_{j}^{i}|\theta_{K}^{1},0\right)$ take the forms defined by Eqs B.1, B.4, B.7, B.10 and B.13.

The likelihood functions for the mixed effects SDE height-diameter models take the following forms:

$$L_{K}^{2}(\theta_{K}^{2})=\prod_{i=1}^{M}\prod_{j=1}^{n_{i}}\int_{-\infty }^{+\infty }f_{K}\left(h_{j}^{i},d_{j}^{i}|\theta_{K}^{1},\phi_{i}\right)\cdot p(\phi_{i}|\sigma_{\phi })\cdot d\phi_{i},\;K\in \left\{V,G3,G4,B,G\right\},$$ (C.3)

and the log-likelihood function is:

$$LL_{K}^{2}(\theta_{K}^{2})=\sum_{i=1}^{M}\int_{-\infty }^{+\infty }\sum_{j=1}^{n_{i}}\ln\left(f_{K}\left(h_{j}^{i},d_{j}^{i}|\theta_{K}^{1},\phi_{i}\right)\right)+\ln\left(p(\phi_{i}|\sigma_{\phi })\right)\cdot d\phi_{i},\;K\in \left\{V,G3,G4,B,G\right\},$$ (C.4)

where $\theta_{K}^{2}$ are fixed effects parameters (the same for all plots) and *ϕ*_*i*_ are random effects (plot specific), which are assumed to follow a normal distribution with *0* mean and constant variance $\sigma_{\phi }^{2}$, and *p*(*ϕ*_*i*_|*σ*_*ϕ*_) is the normal density of the random effects.

The integral in Eq C.4 does not have a closed form solution. Because analytic expression for the integrand in Eq C.4 is known, the Laplace method [39], [40] may be used. Let us define a function *g*:*R*→*R* as follows:

$$g_{K}\left(\phi_{i}|\theta_{K}^{2}\right)=\sum_{j=1}^{n_{i}}\ln\left(f_{K}\left(h_{j}^{i},d_{j}^{i}|\theta_{K}^{1},\phi_{i}\right)\right)+\ln\left(p(\phi_{i}|\sigma_{\phi })\right),\;i=1,\;2,\ldots ,M,\;K\in \left\{V,G3,G4,B,G\right\}.$$ (C.5)

The Laplace approximation to $\int_{-\infty }^{+\infty }e^{g_{K}\left(\phi_{i}|\theta_{K}^{2}\right)}\cdot d\phi_{i}$, *i* = 1,2,…,*M*, *K* ∈ {*V*,*G*3,*G*4,*B*,*G*} is based on a second-order Taylor series expansion about mode ${\hat{\phi }}_{i}$, *i* = 1,2,…,*M*:

$$\int_{-\infty }^{+\infty }e^{g_{K}\left(\phi_{i}|\theta_{K}^{2}\right)}\cdot d\phi_{i}\approx e^{g_{K}\left({\hat{\phi }}_{i}|\theta_{K}^{2}\right)}\int_{-\infty }^{+\infty }\exp\left(\frac{1}{2}\frac{\partial^{2}g_{K}\left({\hat{\phi }}_{i}|\theta_{K}^{2}\right)}{\partial^{2}\phi_{i}}{\left(\phi_{i}-{\hat{\phi }}_{i}\right)}^{2}\right)\cdot d\phi_{i},\;K\in \left\{V,G3,G4,B,G\right\},$$ (C.6)

where ${\hat{\phi }}_{i}$ is the global max of $g_{K}\left(\phi_{i}|\theta_{K}^{2}\right)$ and the root of:

$$\frac{\partial g_{K}\left(\phi_{i}|\theta_{K}^{2}\right)}{\partial \phi_{i}}=0,\;i=1,2,\ldots ,M,\;K\in \left\{V,G3,G4,B,G\right\}.$$ (C.7)

Then, the Laplace approximation of $\ln\left(\int_{-\infty }^{+\infty }e^{g_{K}\left(\phi_{i}|\theta_{K}^{2}\right)}\cdot d\phi_{i}\right)$, *i* = 1,2,…,*M*, *K* ∈ {*V*,*G*3,*G*4,*B*,*G*} takes the following form:

$$\ln\left(\int_{-\infty }^{+\infty }e^{g_{K}\left(\phi_{i}|\theta_{K}^{2}\right)}\cdot d\phi_{i}\right)\approx g_{K}\left({\hat{\phi }}_{i}|\theta_{K}^{2}\right)+\frac{1}{2}\ln\left(2\pi \right)-\frac{1}{2}\ln\left(-\frac{\partial^{2}g_{K}\left({\hat{\phi }}_{i}|\theta_{K}^{2}\right)}{\partial^{2}\phi_{i}}\right),$$ (C.8)

where:

$${\hat{\phi }}_{i}=\underset{\phi_{i}}{\arg\;\max}\;g_{K}\left(\phi_{i}|\theta_{K}^{2}\right),\;i=1,\;2,\;\ldots ,\;M,\;K\in \left\{V,G3,G4,B,G\right\}.$$ (C.9)

The log-likelihood function for the mixed-effects SDEs height-diameter models is approximately given by:

$$LL_{K}^{2}(\theta_{K}^{2})\approx \sum_{i=1}^{M}\left(g_{K}\left({\hat{\phi }}_{i}|\theta_{K}^{2}\right)+\frac{1}{2}\ln\left(2\pi \right)-\frac{1}{2}\ln\left(-\frac{\partial^{2}g_{K}\left({\hat{\phi }}_{i}|\theta_{K}^{2}\right)}{\partial^{2}\phi_{i}}\right)\right),\;K\in \left\{V,G3,G4,B,G\right\}.$$ (C.10)

The maximization of $LL_{K}^{2}(\theta_{K}^{2})$ is a two-step optimization problem. The internal optimization step estimates the ${\hat{\phi }}_{i}$ for ever*y* plot *i* = 1,2,…,*M* with Eq C.9. The external optimization step maximizes $LL_{K}^{2}(\theta_{K}^{2})$ after plugging the ${\hat{\phi }}_{i}$ into Eq C.10.

To assess the asymptotic standard errors of the maximum likelihood estimators for the stochastic height-diameter models, a study of the Fisher [41] information matrix was performed. The approximate asymptotic variance of the approximated maximum likelihood estimators (Eq C.10) was calculated by the inverse of observed Fisher information matrix. By defining the vector${\left(LL_{K}^{s}(\theta^{k})\right)}^{\prime }\equiv \frac{\partial LL_{K}^{s}(\theta_{K}^{s})}{\partial \theta_{K,i}^{s}}$, and the matrix${\left(LL_{K}^{s}(\theta_{K}^{s})\right)}^{\prime \prime }\equiv {\left[\frac{\partial^{2}LL_{K}^{s}(\theta_{K}^{s})}{\partial \theta_{K,i}^{s}\partial \theta_{K,j}^{s}}\right]}^{T}$, s = 1,2, *K* ∈ {*V*,*G*3,*G*4,*B*,*G*}, the observed Fisher information matrix takes the following form:

$$\tilde{I}(\hat{\theta_{K}^{s}})={\left[-\frac{\partial^{2}LL_{K}^{s}(\theta_{K}^{s})}{\partial \theta_{K,i}^{s}\partial \theta_{K,j}^{s}}\right]}^{T}|\theta_{K}^{s}=\hat{\theta_{K}^{s}},\;s=1,2,\;K\in \left\{V,G3,G4,B,G\right\}.$$ (C.11)

The approximate asymptotic standard errors of the fixed effects parameters are defined by the diagonal elements of the matrix${\left[\tilde{I}(\hat{\theta_{K}^{s}})\right]}^{-1}$, s = 1,2, *K* ∈ {*V*,*G*3,*G*4,*B*,*G*} by:

$$SE\left(\hat{\theta_{i}^{}}\right)=\sqrt{{\left[{\tilde{I}}_{ii}(\hat{\theta_{K}^{s}})\right]}^{-1}*\frac{1}{n}}.$$ (C.12)

## Appendix D

### Calibration and stochastic prediction

In the literature on forestry, calibration means that random effects are predicted using a supplementary sample of observations taken from a sampling unit. The tree heights for new stand can be predicted either by using random effects set to zero, or by adding random effects that were predicted from prior observations. When the diameter and height of a sub-sample of trees are known, the predicted random effects are added to the fixed effects parameters to obtain localized parameters for this sub-sample plot.

Let us assume that a sub-sample of *m* trees with height *h*_*j*_ and diameter *d*_*j*_, *j = 1*, *2*, …, *m*, is taken from a new plot. Using height-diameter models defined by Eqs 3–7, the random effect, *ϕ*, for a new stand can be approximately calibrated as follows:

$${\hat{\phi }}_{V}=\frac{1}{m}\sum_{j=1}^{m}\frac{h_{j}-\hat{\alpha }-(1.3-\hat{\alpha })\cdot \exp(-\hat{\beta }\cdot d_{j})}{1-\exp(-\hat{\beta }\cdot d_{j})},$$ (D.1)

$${\hat{\phi }}_{G3}=\frac{1}{m}\left(\sum_{j=1}^{m}\frac{\left(\ln\left(h_{j}\right)-\ln\left(1.3\right)\cdot \exp(-\hat{\beta }\cdot d_{j})\right)\cdot \hat{\beta }}{1-\exp(-\hat{\beta }\cdot d_{j})}-\frac{\hat{\sigma^{2}}}{4}\left(\exp(-\hat{\beta }\cdot d_{j})-1\right)-\hat{\alpha }\right),$$ (D.2)

$${\hat{\phi }}_{G4}=\frac{1}{m}\left(\sum_{j=1}^{m}\frac{\left(\ln\left(h_{j}-\hat{\gamma }\right)-\ln\left(1.3-\hat{\gamma }\right)\cdot \exp(-\hat{\beta }\cdot d_{j})\right)\cdot \hat{\beta }}{1-\exp(-\hat{\beta }\cdot d_{j})}-\frac{\hat{\sigma^{2}}}{4}\left(\exp(-\hat{\beta }\cdot d_{j})-1\right)-\hat{\alpha }\right),$$ (D.3)

$${\hat{\phi }}_{B}=\frac{1}{m}\sum_{j=1}^{m}\left(\frac{\ln\left(h_{j}\right)-\ln\left(1.3\right)}{\ln\left(1-\hat{\gamma }\exp\left(-\hat{\beta }d_{j}\right)\right)-\ln\left(1-\hat{\gamma }\right)}-\hat{\alpha }\right),$$ (D.4)

$${\hat{\phi }}_{G}=\frac{1}{m}\sum_{j=1}^{m}\left(\frac{\ln\left(h_{j}\right)-\ln\left(1.3\right)+\hat{\beta }\left(d_{j}-0.001\right)}{\ln\left(d_{j}\right)-\ln\left(0.001\right)}-\hat{\alpha }\right),$$ (D.5)

where $\hat{\alpha },\;\hat{\beta },\hat{\gamma }$ are the parameter estimates calculated using the approximated maximum likelihood procedure (Eq C.10). The height of another tree from the same plot can be predicted by adding the random effect calibrated by Eqs D.1–D.5 to the fixed effects parameter $\hat{\alpha }$, respectively. The random effects height distribution models explain much more variability than the fixed effects models and provide better height-diameter model fitting. The calibrated height distribution models allow accurate results to be obtained with a very small sampling effort, making this approach highly effective and useful.

Mixed effects models incorporate the variability between plots using the expression of the model's parameters in terms of both fixed and random effects. Random effects are conceptually random variables; they can be simulated as such, in terms of utilizing their distribution. To address this, we can also add a random component to the height prediction. This stochastic prediction approach uses distribution functions of random variable, *H*(*d*), and their confidence intervals. The stochastic predictions, *h*_*stoch*,*K*_, *K* ∈ {*V*,*G*3,*G*4,*B*,*G*}, of a tree height can be defined as follows:

$$\hat{h}{\left(d\right)}_{stoch,V}={\hat{m}}_{V}(d)+\Phi_{U}^{-1}\left(0;\hat{\lambda_{V}^{2}}(d)\right)=\Phi_{U}^{-1}\left({\hat{\mu }}_{V}(d);\hat{\lambda_{V}^{2}}(d)\right),$$ (D.6)

$$\hat{h}{\left(d\right)}_{stoch,K}={\hat{m}}_{K}(d)+LN_{U}^{-1}\left(0;\hat{\lambda_{K}^{2}}(d)\right)=LN_{U}^{-1}\left({\hat{\mu }}_{K}(d);\hat{\lambda_{K}^{2}}(d)\right),\;K\in \left\{G3,B,G\right\}$$ (D.7)

$$\hat{h}{\left(d\right)}_{stoch,G4}={\hat{m}}_{G4}(d)+LN_{U}^{-1}\left(0;\hat{\lambda_{G4}^{2}}(d)\right)=\hat{\gamma }+LN_{U}^{-1}\left({\hat{\mu }}_{G4}(d);\hat{\lambda_{G4}^{2}}(d)\right),$$ (D.8)

where ${\hat{m}}_{K}(d)$, *K* ∈ {*V*,*G*3,*G*4,*B*,*G*} is the estimated trend of the mean (calculated using Eqs B.16, B.18, B.20, B.22 and B.24) of the tree height; and $\Phi_{U}^{-1}\left({\hat{\mu }}_{V}(d);\hat{\lambda_{V}^{2}}(d)\right)$ ($LN_{U}^{-1}\left({\hat{\mu }}_{K}(d);\hat{\lambda_{K}^{2}}(d)\right)$, *K* ∈ {*G*3,*G*4,*B*,*G*}) is the inverse of the normal (the lognormal) distribution with a mean of ${\hat{\mu }}_{K}(d)$, *K* ∈ {*V*,*G*3,*G*4,*B*,*G*} defined by Eqs B.2, B.5, B.8, B.11 and B.14, and a variance of $\hat{\lambda_{K}^{2}}(d)$, *K* ∈ {*V*,*G*3,*G*4,*B*,*G*} defined by Eqs B.3, B.6, B.9, B.12 and B.15, for a uniform random variable, *U*, in the interval (0;1).
